# The Dilemma of Stump Appendicitis: A Case Report

**DOI:** 10.7759/cureus.104117

**Published:** 2026-02-23

**Authors:** Avinash Kumar, Maninder K Chhabra, Puneet Chhibber

**Affiliations:** 1 General Surgery, Deen Dayal Upadhyay Hospital, New Delhi, IND

**Keywords:** appendix stump, faecal peritonitis, recurrent appendicitis, retrocaecal appendix, stump

## Abstract

Stump appendicitis is an uncommon but clinically significant delayed complication of appendectomy, arising from inflammation of remnant appendiceal tissue after the initial procedure. Because most clinicians do not consider appendicitis in a post-appendectomy patient, stump appendicitis frequently results in diagnostic delay and increased morbidity.

We report the case of a 27-year-old man from a northern state of India who presented with features of generalized peritonitis one year after undergoing open appendectomy for acute appendicitis. Radiological evaluation suggested inflammation but failed to identify the specific pathology. Under exploratory laparotomy, a 3 cm, inflamed, and perforated appendiceal stump was identified and excised. The patient made an uneventful recovery.

This case highlights the importance of maintaining a high index of suspicion for stump appendicitis in patients with previous appendectomy presenting with acute abdomen and emphasizes the need for meticulous surgical technique during the primary procedure.

## Introduction

Appendectomy is among the most commonly performed emergency surgical operations worldwide and is widely regarded as the definitive management for acute appendicitis. Initially carried out through an open approach and now increasingly via laparoscopy, appendectomy has established a reputation for safety, technical simplicity, and a low rate of complications. Nevertheless, despite its widespread practice, the procedure is not entirely free from pitfalls, with stump appendicitis being one of the rarest yet most clinically challenging delayed complications [1].

Stump appendicitis

Stump appendicitis denotes inflammation of the remaining appendiceal tissue following an appendectomy. In 1945, Rose first reported recurrent appendicitis in a patient with a prior history of appendectomy [2]. Subsequently, in 1949, Baumgardner was the first to introduce the term “stump appendicitis” [3].

Epidemiology and risk factors

The reported incidence is low, estimated at approximately 1 in 50,000 cases; however, it may be considerably underreported because of misdiagnosis or low clinical suspicion [4]. Notably, stump appendicitis can occur months to decades after the initial appendectomy, with cases documented even 50 years later. Although stump appendicitis may develop following either open or laparoscopic appendectomy, its presentation is frequently delayed due to the assumption that the appendix has already been removed.

The pathogenesis of stump appendicitis may be attributed to several factors that interfere with the complete excision of the appendix. Anatomical variations, such as a retrocecal or subserosal appendix and a duplicated appendix, can complicate identification of the true appendiceal base [5]. In addition, surgical circumstances, including severe local inflammation, edema, adhesions, or concern for cecal injury, may prompt surgeons to leave a longer residual stump, thereby increasing the risk of subsequent obstruction, inflammation, and perforation [6]. Inadequate visualization during the procedure, particularly in emergency settings, often performed by junior surgeons, further contributes to the risk.

Diagnostic considerations

Because of its rarity, limited awareness, and clinical presentation that mimics acute appendicitis, stump appendicitis presents a diagnostic dilemma [7]. Radiological modalities may also fail to detect the inflamed remnant, particularly when the stump is very short. Failure to identify this condition at an early stage can lead to complications such as perforation, pyoperitoneum, gangrene, or intra-abdominal abscess [8]. Therefore, awareness of the possibility of stump appendicitis is essential for clinicians when evaluating post-appendectomy patients presenting with an acute abdomen.

Rationale

This case report describes a young adult male who presented with an acute abdomen one year following an open appendectomy. The clinical, radiological, intra-operative findings, and postoperative outcomes are discussed along with a review of the relevant literature, highlighting key diagnostic and preventive considerations.

## Case presentation

A 27-year-old man from a low socioeconomic background from the northern region of India presented to the emergency department with a 3-day history of abdominal pain, progressive vomiting, and anorexia, followed by fever for 1 day. He described the onset of pain as sudden, beginning in the periumbilical region before becoming diffuse across the entire abdomen. The pain was colicky in nature, non-radiating, and mildly relieved by analgesics. The vomiting consisted of two to three episodes of non-projectile, non-bilious emesis containing gastric contents. He denied any history of diarrhoea, constipation, urinary symptoms, or hematuria.

The patient had undergone an open appendectomy one year earlier for acute appendicitis; his postoperative course had been uneventful at that time. He had no known comorbidities, such as diabetes mellitus, hypertension, tuberculosis, or asthma, and did not use tobacco or other substances.

**Figure 1:**
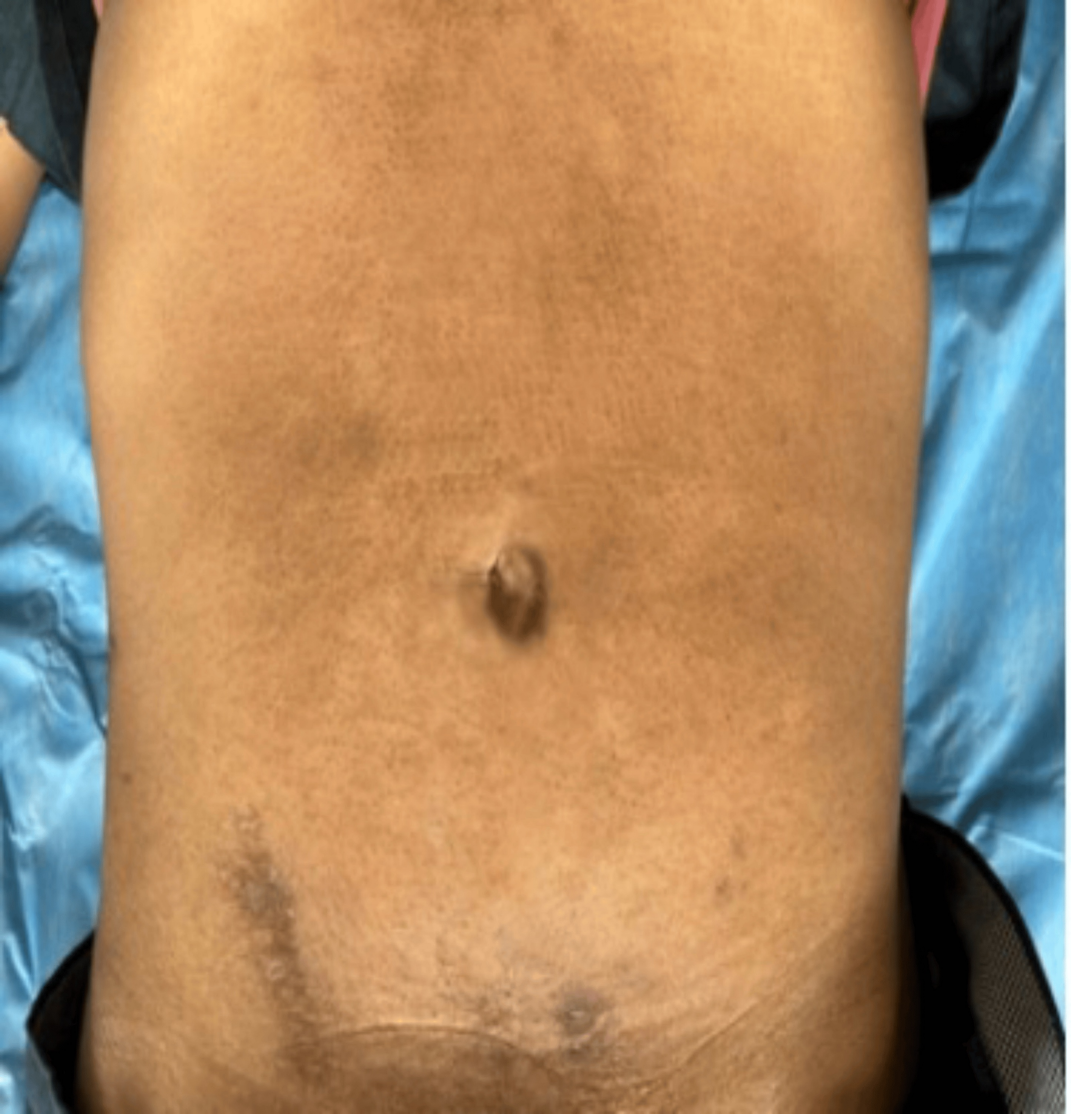
Healed scar of previous appendectomy in the right iliac fossa

On examination, the patient was alert and oriented but in obvious discomfort. He was afebrile at presentation, with a pulse rate of 110/min, blood pressure of 100/64 mmHg, respiratory rate of 22/min, and oxygen saturation of 98% on room air. Physical examination revealed a healed 5-cm scar in the right iliac fossa, as shown in Figure 1. Abdominal palpation elicited diffuse tenderness with generalized guarding and rebound tenderness. Bowel sounds were absent, and the per rectal examination revealed no abnormalities.

**Figure 2:**
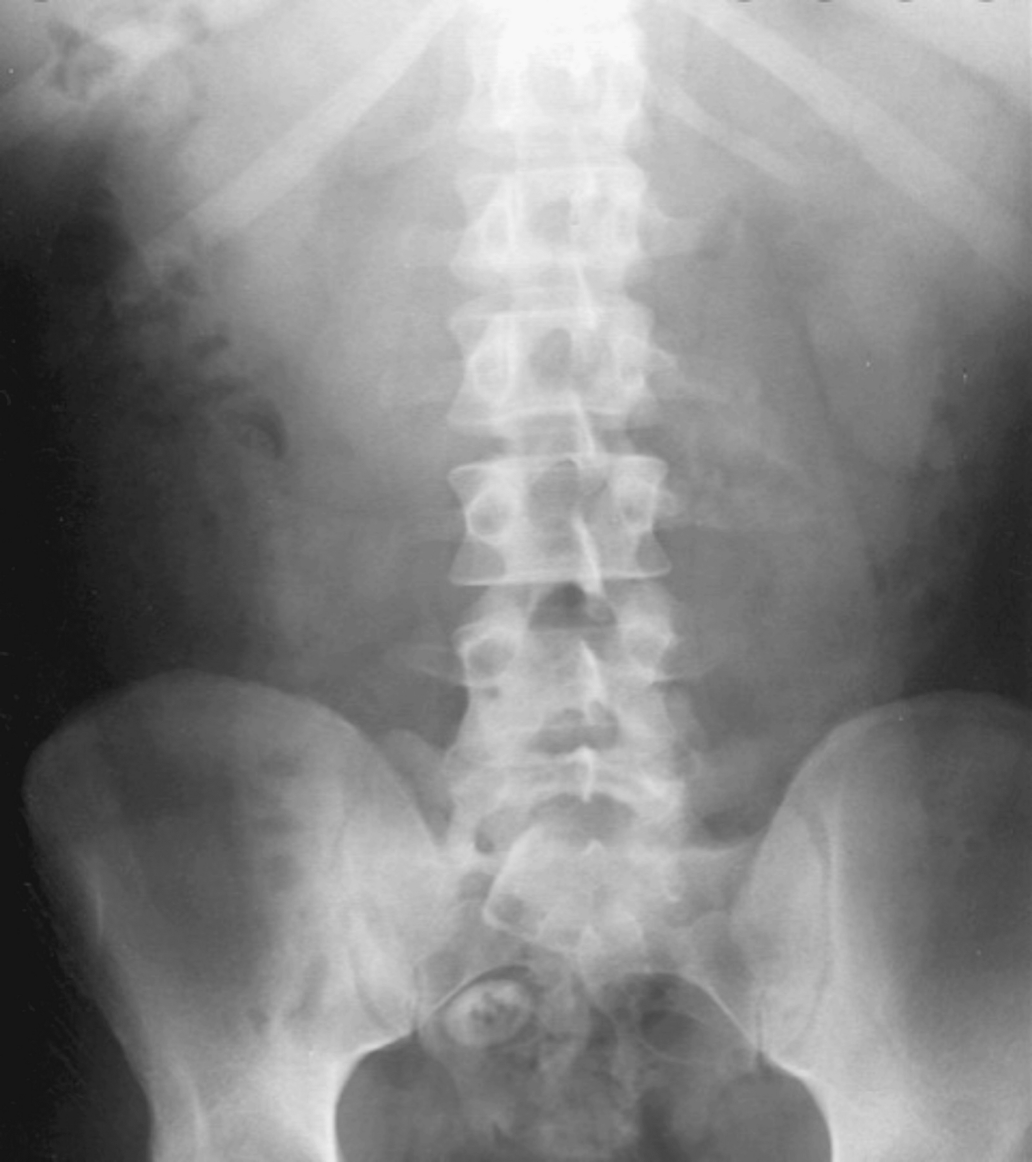
X-ray abdomen of the patient showing no obvious abnormal dilatation of the bowel loops or any free air

Laboratory investigations showed a leucocytosis of 14,000/cmm with neutrophilic predominance; all the hematological and biochemical lab results have been mentioned in Tables 1-2. Renal and liver function parameters were within normal limits. X-ray abdomen showed no free air under the diaphragm or abnormal air-fluid levels, as shown in Figure 2. Ultrasonography of the abdomen revealed a thickened terminal ileum, adjacent inflamed mesentery, and free fluid in the peritoneal cavity. A diagnostic percutaneous tap yielded purulent fluid, confirming the presence of pyoperitoneum.

**Table 1:** Biochemical laboratory results of the patient with the normal reference range mg/dL - milligram per deciliter; mmol/L - millimole per liter; U/L - unit per liter

Test Parameter	Patient Value	Normal Range
Random Blood Sugar (RBS)	87 mg/dL	70-140 mg/dL
Blood Urea	25 mg/dL	15-45 mg/dL
Serum Creatinine	0.4 mg/dL	0.6-1.2 mg/dL
Serum Sodium (Na⁺)	138 mmol/L	135-145 mmol/L
Serum Potassium (K⁺)	3.5 mmol/L	3.5-5.0 mmol/L
Total Serum Bilirubin	0.4 mg/dL	0.2-1.2 mg/dL
Serum Amylase	45 U/L	30-110 U/L
Serum Lipase	128 U/L	0-160 U/L

**Table 2:** All the hematological laboratory reports of the patient with the normal reference range g/dL - gram per deciliter; cmm - cubic millimeter

Test Parameter	Patient Value	Normal Range (Adult)
Hemoglobin (Hb)	14.2 g/dL	Male: 13-17 g/dL Female: 12-15 g/dL
Total Leukocyte Count (TLC)	14,000 /cmm	4,000 – 11,000 /cmm
Differential Leukocyte Count (DLC)		
• Neutrophils (P)	88%	40-75%
• Lymphocytes (L)	9%	20-45%
• Monocytes (M)	2%	2-10%
Platelet Count	3.8 lakh/cmm	1.5-4.5 lakh/cmm

**Figure 3:**
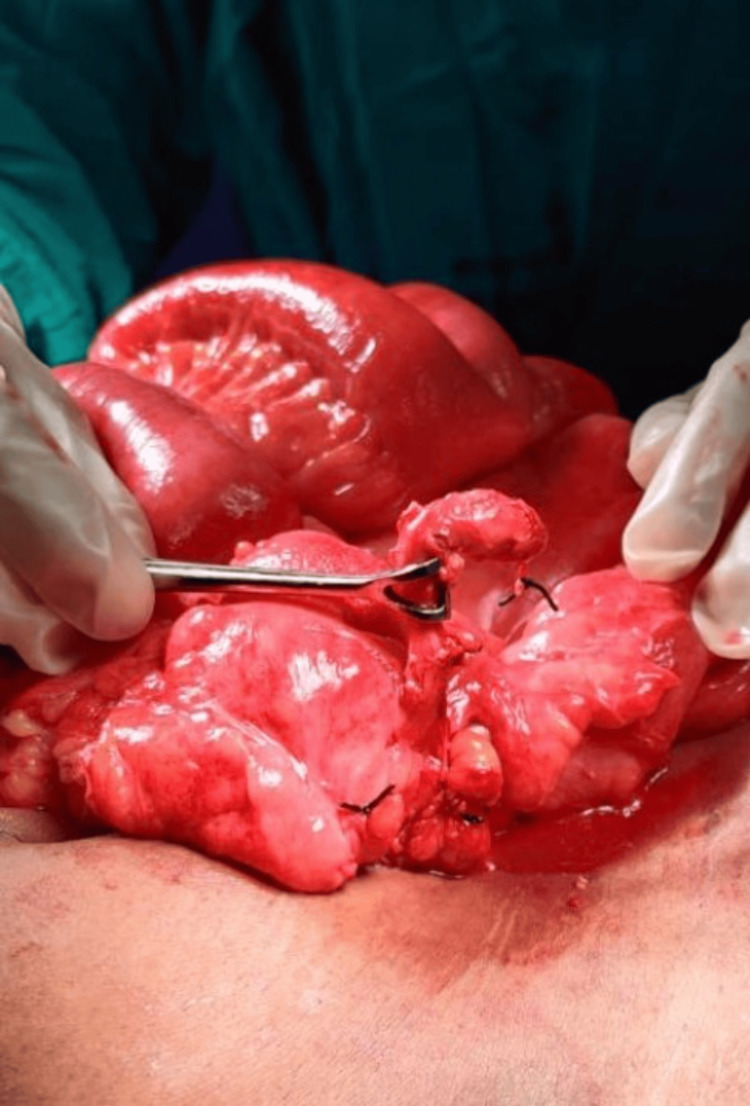
Inflamed appendiceal stump with perforation at the tip

**Figure 4:**
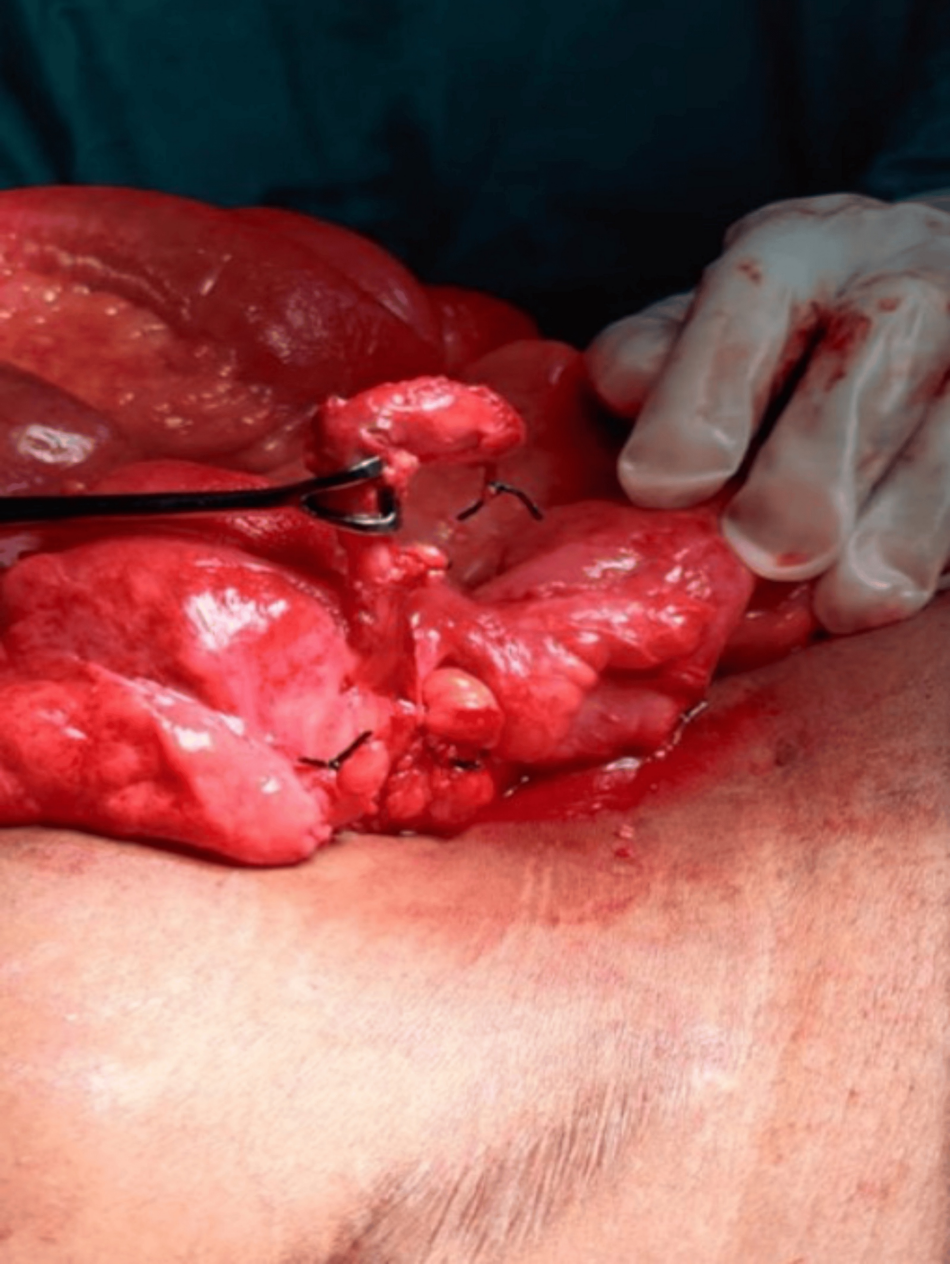
Inflamed appendiceal stump

Given these findings, the patient was resuscitated and taken for emergency exploratory laparotomy through a midline incision. Approximately 500 ml of purulent fluid was encountered in the pelvis and paracolic gutters. The bowel loops were inflamed but intact, with no evidence of perforation or obstruction. Careful exploration revealed a 3-cm, inflamed appendiceal stump, perforated at its tip, which is visible in Figures 3-4. The cecal base was healthy. A completion appendectomy was performed, followed by thorough peritoneal lavage and drainage.

Postoperative recovery was uneventful. The patient resumed oral intake on postoperative day 2, and the abdominal drain was removed on postoperative day 3. He was discharged on postoperative day 6. Histopathological examination of the excised residual stump confirmed features of acute appendicitis. Pus culture demonstrated growth of *Klebsiella*, sensitive to amikacin, imipenem, and piperacillin.

## Discussion

Overview of stump appendicitis

Stump appendicitis, although uncommon, is a condition associated with potentially serious consequences if not diagnosed in a timely manner. The concept is not new; however, its low incidence and misleading clinical setting often result in diagnostic delays. The condition is defined as inflammation of the residual appendiceal tissue remaining after appendectomy. Reports suggest that residual stumps measuring as short as 0.5 cm and as long as 6.5 cm have been implicated, with a mean stump length of approximately 3 cm described in certain series [6].

Findings in the present case

In our patient, the residual stump measured approximately 3 cm and was completely inflamed, resulting in perforation and pyoperitoneum. This underscores the critical importance of adequate visualization and precise identification of the appendiceal base during the initial appendectomy [9]. Incomplete excision of the appendix may occur due to difficulty in accessing the base, the presence of edematous tissue, severe inflammation, or adhesions. In emergency surgical settings, time constraints and limited experience among junior surgeons may further increase the risk of leaving behind a longer residual stump [6].

Anatomical and surgical risk factors

Anatomical variations also contribute to the risk. A retrocecal or subserosal position of the appendix may obscure the base during dissection, leading to inadvertent retention of a portion of the appendix [6]. In addition, an entirely or partially retrocecal appendix, where the base lies retrocecal or a segment of the appendiceal shaft is retrocecal, causing the tip to turn back and be clearly visualized intraperitoneally, may result in misidentification of the portion that disappears into the retrocecal area, with false transection of the base and a residual stump left behind [10]. Rarely, a duplicated appendix may also be responsible for prolonged symptoms or recurrent inflammation [11].

Diagnostic challenges

Diagnostic evaluation of stump appendicitis remains challenging. Clinical examination may demonstrate classical features of appendicitis; however, this often contradicts the patient’s previous history of appendectomy. Ultrasonography is operator dependent, and a short residual stump may be overlooked or misinterpreted. Computed tomography (CT), particularly contrast-enhanced CT, has been reported to be more sensitive and may demonstrate typical findings such as peri-caecal inflammation, fluid collections, the arrowhead sign, and caecal wall thickening [5]. Nevertheless, even CT may produce equivocal findings, especially in the presence of extensive peritonitis that obscures localized inflammatory changes. In the present case, contrast-enhanced computed tomography (CECT) failed to clearly visualize the stump, highlighting the diagnostic difficulty. The diagnostic tap yielding pus ultimately guided the decision to proceed with exploratory surgery, which remains the gold standard in patients with generalized peritonitis [6].

Treatment approaches

Although there is evidence that uncomplicated acute appendicitis may be managed conservatively with antibiotics, no such robust evidence exists for stump appendicitis. A literature review published in 2011 reported that among 40 cases of stump appendicitis, all patients underwent surgical intervention, and only 33% were treated laparoscopically. In patients for whom operative management may not be an appropriate option, conservative treatment with intravenous antibiotics has been described [12,13].

The management of stump appendicitis consists of definitive excision of the residual appendiceal stump--a completion appendectomy--performed either through an open or laparoscopic approach, depending on the patient’s clinical stability and the surgeon’s expertise. There are three basic techniques for managing the appendiceal stump, namely, simple ligation, ligation with inversion, and inversion without ligation; however, no consensus exists regarding the optimal method [14]. In severely inflamed or late-presenting cases, more extensive procedures such as ileocecal resection may be necessary, although this is uncommon [6].

Preventive considerations

Preventive strategies primarily focus on surgical technique. The concept of the critical view of safety, well-established in laparoscopic cholecystectomy, has also been recommended for laparoscopic appendectomy. This approach entails meticulous dissection to clearly identify the appendiceal base, ensuring that only the true base at the appendiceal-caecal junction is ligated and that the residual stump length is maintained within 3-5 mm. Ensuring appropriate training and supervision of junior surgeons, particularly in emergency settings, is essential to prevent incomplete excision of the appendix [9].

Some authors have advocated routine inversion of the stump in all cases following appendectomy as a means of reducing the incidence of stump appendicitis; however, others consider this unnecessary provided that an appendiceal stump measuring no more than 3 mm in length is left behind [15].

Clinical implications

This case reinforces the need for clinicians to maintain a high index of suspicion when evaluating post-appendectomy patients presenting with abdominal pain, vomiting, and fever. Failure to recognize stump appendicitis may result in serious complications, including perforation, gangrene, abscess formation, or generalized peritonitis, all of which can markedly increase morbidity and prolong hospitalization.

## Conclusions

Stump appendicitis remains a significant diagnostic challenge because of its rarity and the misleading clinical history of a prior appendectomy. This case highlights the importance of maintaining a high index of clinical suspicion, performing timely and appropriate imaging, and instituting prompt surgical intervention in order to prevent potentially serious complications. Furthermore, ensuring complete excision of the appendix during the primary procedure and strict adherence to sound surgical principles are essential measures to minimize the risk of this complication and improve patient outcomes.
